# Tooth Preservation Through Root-End Surgery in a 15-Year-Old Patient With Refractory Periapical Pathology: A Case Report

**DOI:** 10.7759/cureus.111362

**Published:** 2026-06-23

**Authors:** Rutuja B Salunkhe, N.D. Shashikiran, Namrata Gaonkar, Sandisha S Sudrik, Ruchira R Sawant

**Affiliations:** 1 Department of Paediatric and Preventive Dentistry, School of Dental Sciences, Krishna Vishwa Vidyapeeth, Karad, IND; 2 Department of Paediatric and Preventive dentistry, School of Dental Sciences, Krishna Vishwa Vidyapeeth, Karad, IND

**Keywords:** apicectomy, dental trauma, endodontic surgery, guided tissue regeneration, periapical lesion, platelet-rich fibrin, root-end surgery, sticky bone

## Abstract

Persistent periapical lesions associated with traumatized immature or mature permanent teeth can pose a significant therapeutic challenge when conventional nonsurgical endodontic treatment fails to achieve healing. Surgical endodontic intervention serves as a predictable alternative for preserving natural dentition while eliminating persistent periapical pathology. This case report describes the management of a large refractory periapical lesion associated with maxillary anterior teeth in a 15-year-old patient with a history of dental trauma. Despite five months of intracanal medicament therapy following root canal treatment, the lesion failed to resolve clinically and radiographically. Cone-beam computed tomography (CBCT) revealed a well-defined periapical radiolucency with cortical plate perforation. Surgical management included apicectomy, periapical curettage, laser-assisted debridement, platelet-rich fibrin (PRF)-assisted bone regeneration using sticky bone, and placement of a guided tissue regeneration (GTR) membrane. Histopathological examination confirmed chronic nonspecific inflammation. Clinical and radiographic follow-up demonstrated satisfactory healing and resolution of symptoms. This case highlights the effectiveness of contemporary endodontic microsurgical principles combined with regenerative techniques in preserving natural teeth affected by persistent periapical pathology.

## Introduction

Traumatic injuries involving the maxillary anterior teeth are common during childhood and adolescence and may result in pulpal necrosis and subsequent periapical pathosis long after the initial traumatic event [1]. The management of teeth with pulpal necrosis and associated periapical lesions typically involves conventional nonsurgical root canal treatment, which has demonstrated high success rates in eliminating intracanal infection and promoting periapical healing [2]. However, certain lesions may persist despite adequate chemomechanical preparation and intracanal medicament therapy due to factors such as extraradicular infection, true cyst formation, foreign body reactions, or anatomical complexities that limit complete disinfection of the root canal system [2,3].

When nonsurgical treatment fails to achieve satisfactory clinical and radiographic healing, endodontic surgery becomes a valuable treatment option for preserving natural dentition [3]. Contemporary apical surgery focuses on complete removal of pathological tissue, root-end resection, effective apical sealing, and regeneration of the surrounding periapical structures, resulting in significantly improved treatment outcomes compared with traditional surgical approaches [3,4].

Recent advances in regenerative dentistry have further enhanced the prognosis of large periapical defects. Platelet-rich fibrin (PRF) serves as an autologous source of growth factors that promote angiogenesis, osteogenesis, and soft tissue healing, while guided tissue regeneration (GTR) membranes help prevent soft tissue migration into osseous defects and facilitate selective tissue regeneration [5,6]. In addition, cone-beam computed tomography (CBCT) has become an indispensable diagnostic tool for evaluating the true extent of periapical lesions, cortical plate involvement, and proximity to adjacent anatomical structures, thereby improving surgical planning and treatment predictability [7].

The present report describes the successful management of a persistent traumatic periapical lesion associated with maxillary anterior teeth in a 15-year-old patient using apicectomy, laser-assisted debridement, platelet-rich fibrin-based sticky bone grafting, and guided tissue regeneration following unsuccessful nonsurgical endodontic treatment.

## Case presentation

A 15-year-old patient reported to the Department of Pediatric and Preventive Dentistry with a chief complaint of pain in the upper front tooth region for two months. The patient was apparently asymptomatic until two months prior to presentation, when pain developed in the maxillary anterior region. The pain was dull, throbbing, continuous, and localized in nature. Temporary relief was obtained following the use of analgesic medication. The patient also reported recurrent episodes of swelling in the same region during the previous two months. The swelling gradually increased in size and was occasionally painful. There was no history of fever, pus discharge, or associated systemic symptoms. The patient's dental history revealed trauma to the maxillary anterior region following a fall from a bicycle four years earlier.

Clinical examination

**Figure 1:**
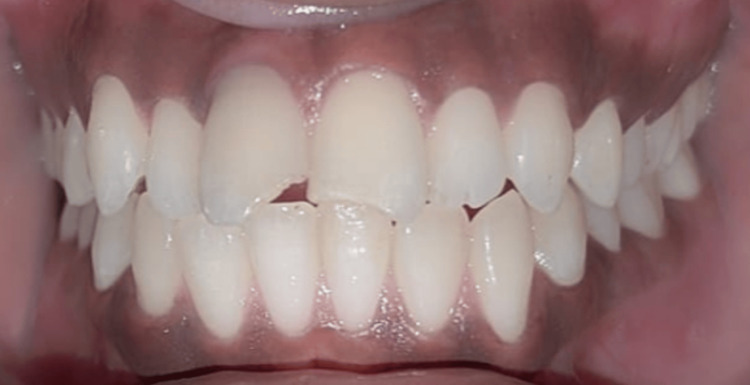
Intraoral examination

Intraoral examination revealed diffuse, soft swelling in the apical region of teeth 11, 12, and 21. The overlying mucosa appeared stretched. Tenderness on vertical percussion was present in relation to teeth 11, 12, and 21 (Figure 1). 

Diagnostic assessment

**Figure 2:**
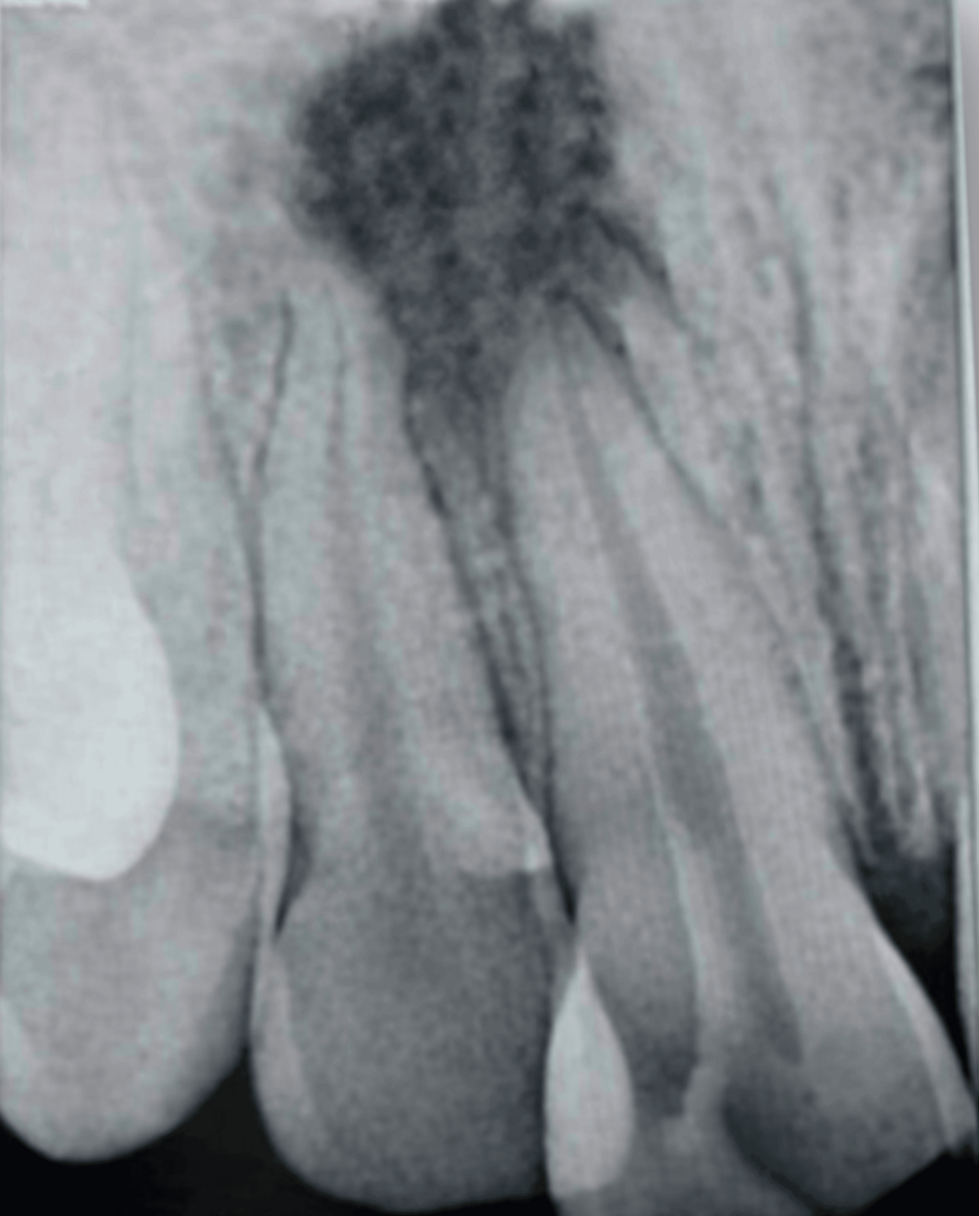
Radiographic examination

A provisional diagnosis of pulpal necrosis with chronic apical abscess was established. Electric pulp testing demonstrated non-vital responses in teeth 11, 12, and 21, while adjacent teeth responded normally. Periapical radiographic examination revealed a well-defined, unilocular, round radiolucent lesion involving the apical regions of teeth 11 and 12 (Figure 2).

**Figure 3:**
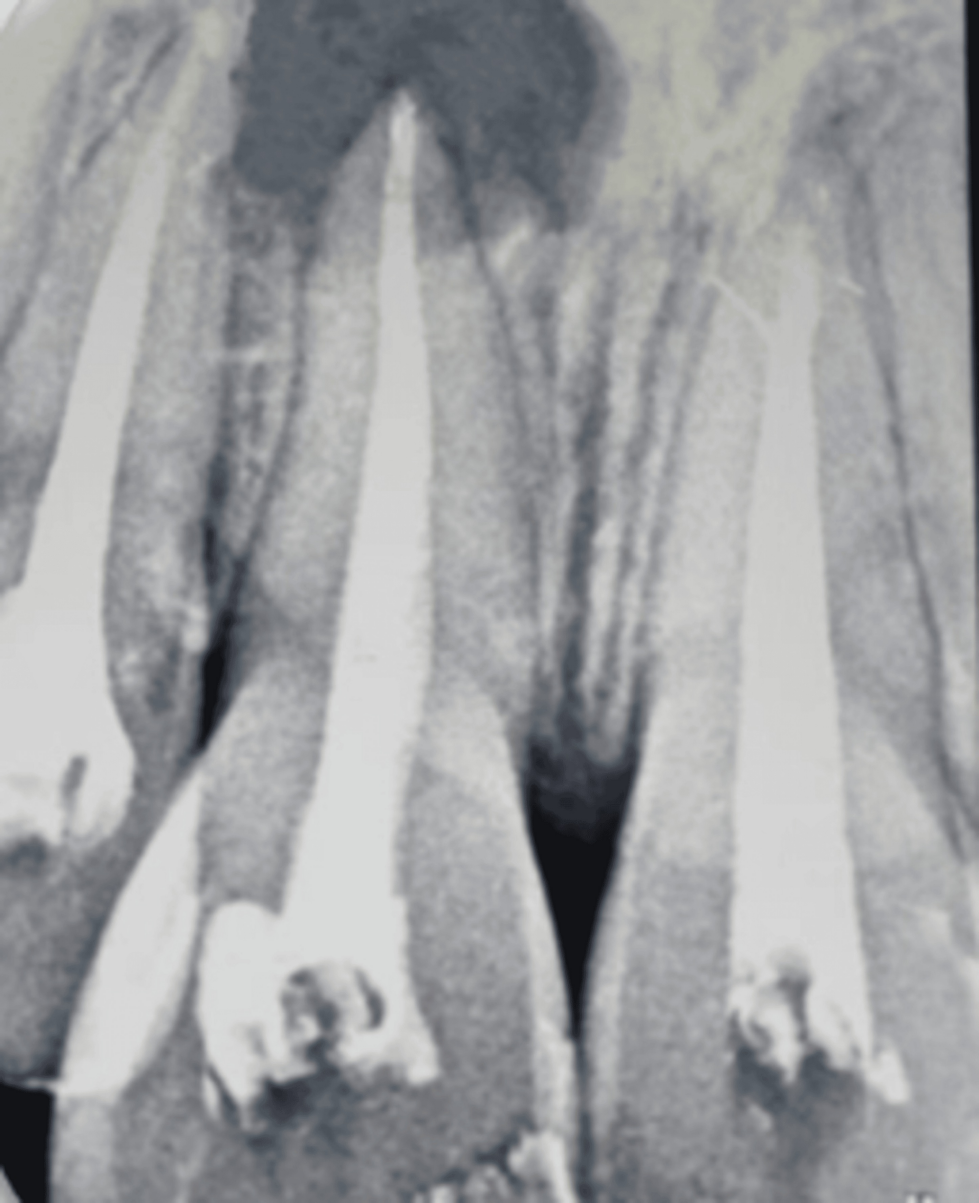
Metapex dressing was given

Based on clinical and radiographic findings, nonsurgical root canal treatment was initiated for teeth 11, 12, and 21. Calcium hydroxide-based intracanal medicament (Metapex) was placed and periodically replaced. Despite repeated intracanal dressings over a five-month period, no significant clinical or radiographic improvement was observed (Figure 3). Because radiographic healing of larger periapical lesions may require 6-12 months or longer after intracanal disinfection, treatment outcomes should be assessed in conjunction with clinical findings rather than radiographic appearance alone [2]. 

Therefore, surgical intervention was planned. Routine hematological investigations were within normal limits. CBCT imaging was obtained to evaluate the extent of the lesion and its relationship to adjacent anatomical structures, including the nasal floor.

**Figure 4:**
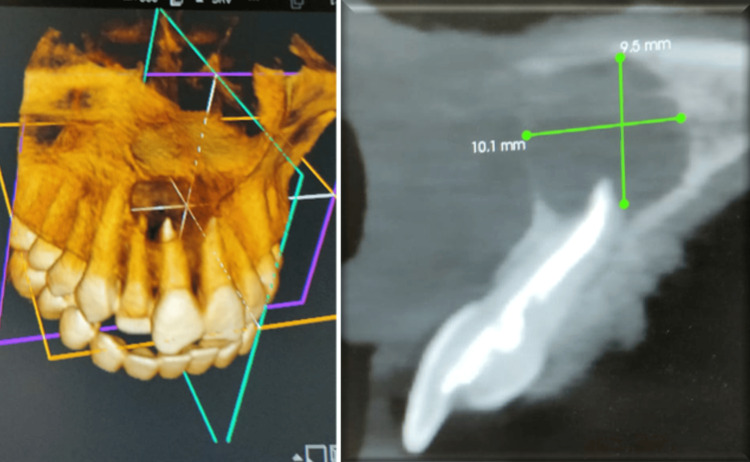
CBCT examination CBCT: cone-beam computed tomography.

CBCT examination revealed a well-defined periapical radiolucency measuring approximately 10.1 mm mediolaterally and 9.5 mm anteroposteriorly. The lesion extended from the mesial aspect of tooth 13 to the mesial aspect of tooth 11. Axial sections demonstrated expansion and perforation of both the labial and palatal cortical plates in relation to teeth 11 and 12 (Figure 4). 

**Figure 5:**
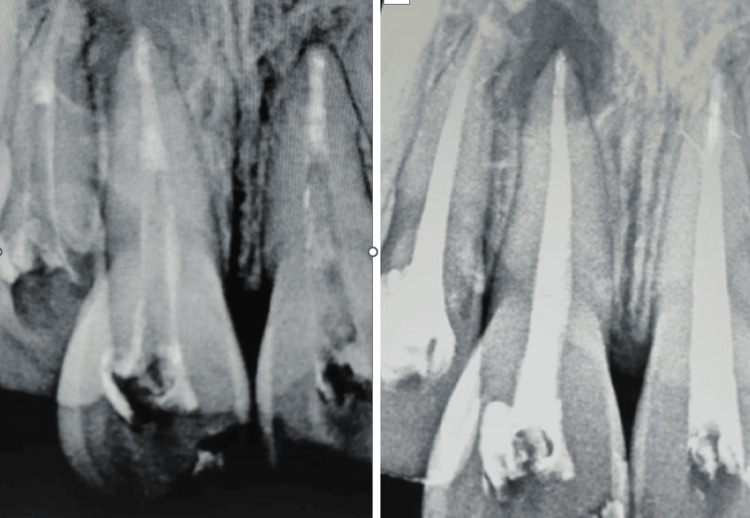
An apical plug of 6 mm was placed using mineral trioxide aggregate (MTA) followed by completion of the coronal obturation done

One day before surgery, orthograde obturation of the root canals was completed. A 6-mm mineral trioxide aggregate (MTA) apical plug was placed, followed by completion of coronal obturation (Figure 5).

Surgical procedure

**Figure 6:**
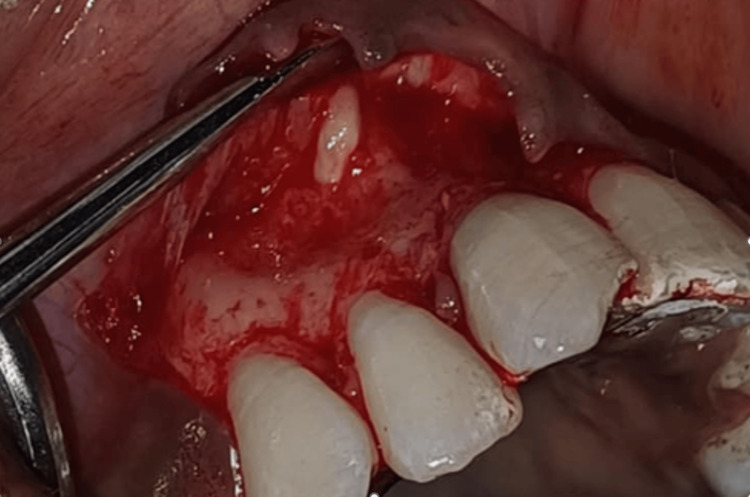
Full-thickness mucoperiosteal flap is raised

Written informed consent was obtained from the patient's parent before treatment. Under local anesthesia and strict aseptic conditions, a full-thickness mucoperiosteal flap was designed. A crevicular incision with an anterior releasing incision was placed, preserving the interdental papillae. The flap was carefully reflected to expose the surgical site (Figure 6).

**Figure 7:**
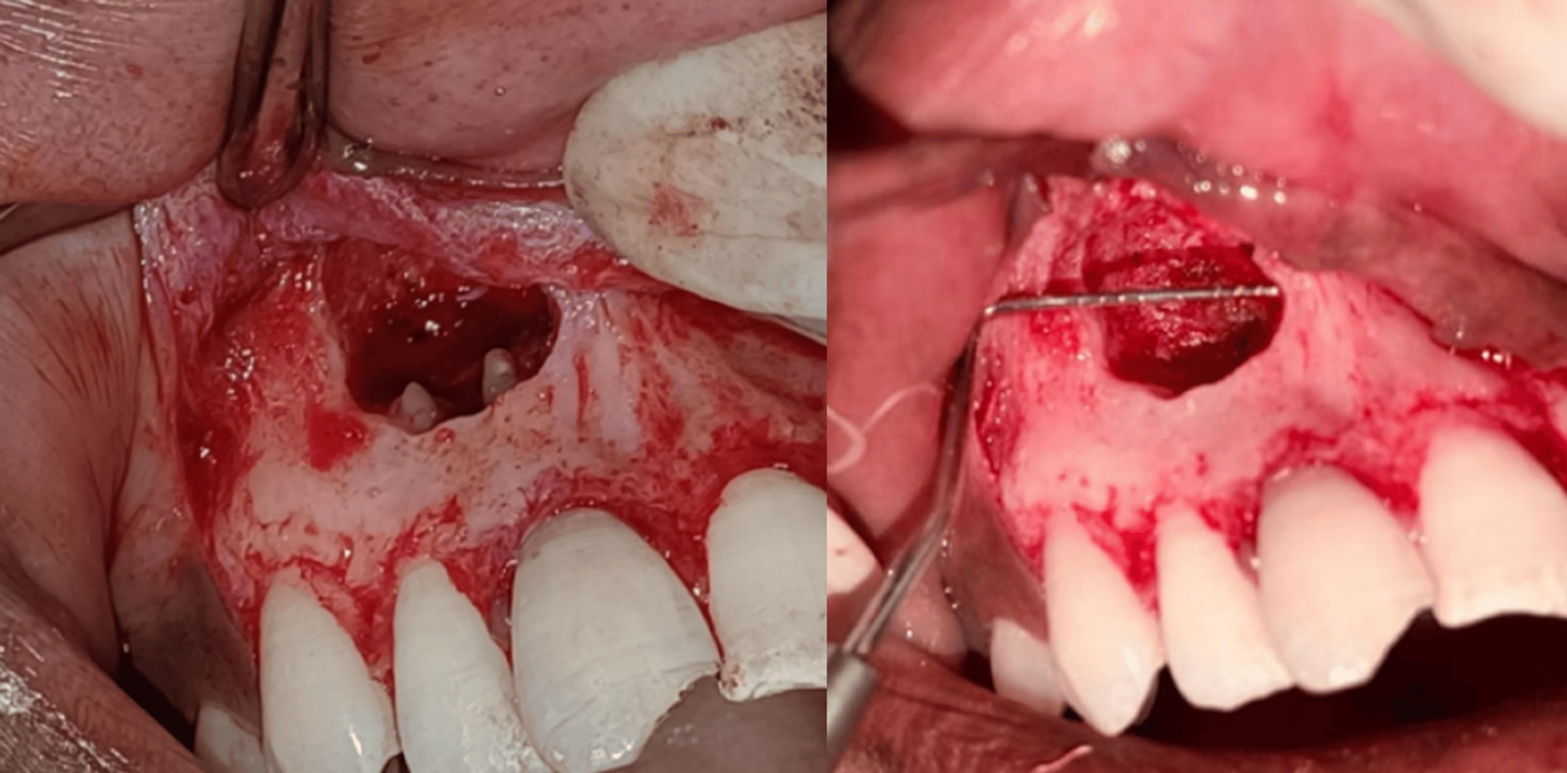
Root resection of 3 mm

The thin and expanded buccal cortical bone was accessed using a round bur to create an oval bony window. Following exposure of the lesion, the cystic lining was identified and carefully separated from the surrounding tissues. Thorough curettage of the pathological tissue was performed. Root-end resection of approximately 3 mm was carried out perpendicular to the long axis of the roots with a 0° bevel (Figure 7). Additional debridement of the periapical region was performed using Lucas curettes. Laser-assisted disinfection and decontamination were carried out using a dual-wavelength laser (640 nm and 810 nm combination mode). The use of laser therapy minimized intraoperative bleeding and enhanced visibility during surgery. 

Regenerative procedure

**Figure 8:**
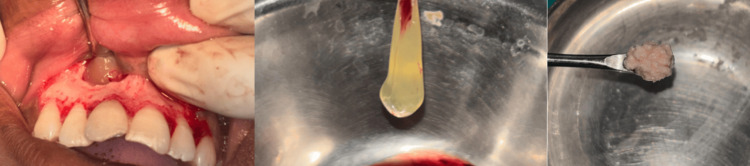
The PRF components were combined with xenograft particles to prepare sticky bone. This regenerative graft material was placed within the osseous defect PRF: platelet-rich fibrin.

Ten milliliters of autologous venous blood was collected and divided equally into two sterile vacuum tubes. One tube underwent centrifugation at 700 rpm for three minutes to obtain injectable platelet-rich fibrin, while the second tube was centrifuged at 3000 rpm for 10 minutes to obtain solid platelet-rich fibrin. The PRF components were combined with xenograft particles to prepare sticky bone. This regenerative graft material was placed within the osseous defect (Figure 8). 

**Figure 9:**
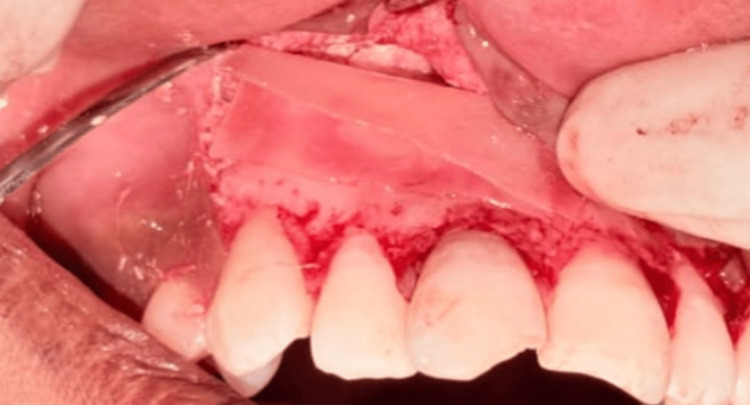
A guided tissue regeneration membrane was adapted over the defect Then flap was repositioned

A guided tissue regeneration membrane was adapted over the defect to facilitate selective regeneration of periodontal and osseous tissues while preventing soft-tissue invasion (Figure 9).

**Figure 10:**
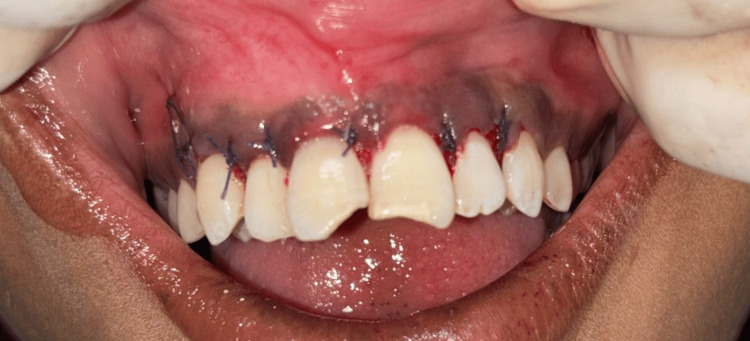
Suture placement

The membrane was carefully adapted over the surgical defect and stabilized using simple interrupted 4-0 Vicryl sutures to ensure secure positioning and promote optimal wound healing (Figure 10).

Postoperative care and follow-up

Postoperative medications included antibiotics, analgesics, and an antimicrobial mouth rinse. The patient was provided with detailed postoperative instructions. Histopathological examination revealed chronic nonspecific inflammatory tissue.

**Figure 11:**
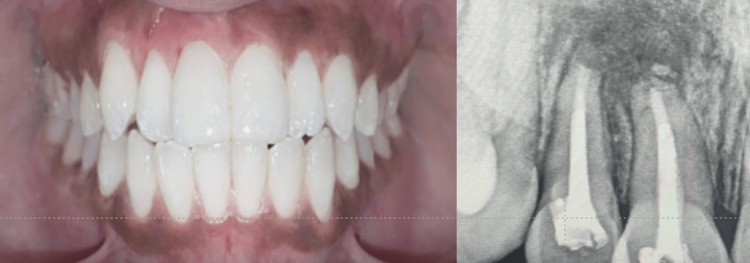
Composite restoration and radiograph at one-month follow-up

The patient was reviewed after seven days. Healing was satisfactory, and the sutures were removed. Following shade selection, definitive composite restoration of the treated teeth was completed. At the one-month follow-up, the patient was asymptomatic, and clinical examination demonstrated satisfactory soft tissue healing with no evidence of swelling or tenderness (Figure 11). 

## Discussion

The development of periapical pathology following traumatic dental injuries is a well-recognized sequela, particularly when pulpal necrosis remains untreated or undiagnosed for an extended period. In the present case, the patient reported a history of trauma to the maxillary anterior region four years prior to presentation. Delayed pulpal necrosis after traumatic injury has been documented in the literature and may manifest clinically as pain, swelling, sinus tract formation, or radiographic evidence of periapical bone destruction several years after the initial injury [8].

Conventional nonsurgical root canal treatment remains the primary treatment modality for teeth with pulpal necrosis and apical periodontitis. Calcium hydroxide-based intracanal medicaments, such as Metapex, are widely used because of their antimicrobial properties and ability to promote periapical healing. Nevertheless, complete resolution of periapical lesions cannot always be achieved through orthograde treatment alone. Persistent lesions may be associated with extraradicular infection, actinomycotic colonization, cholesterol crystal deposition, foreign body reactions, or the presence of true cystic cavities that are self-sustaining and independent of intracanal infection [9,10]. In the present case, repeated intracanal medication over a five-month period failed to produce satisfactory clinical improvement, necessitating surgical intervention.

Accurate assessment of lesion size and anatomical involvement is essential before undertaking endodontic surgery. Conventional periapical radiographs provide only two-dimensional information and may underestimate the true extent of osseous destruction. The use of CBCT in the present case enabled detailed visualization of lesion dimensions and confirmed perforation of both the labial and palatal cortical plates. Previous studies have demonstrated that CBCT improves diagnostic accuracy, facilitates surgical planning, and assists in evaluating the relationship of lesions to adjacent anatomical structures, thereby improving treatment predictability [11].

The decision to perform apicectomy was based on the persistence of symptoms and radiographic pathology despite adequate root canal therapy. Modern endodontic microsurgery emphasizes conservative osteotomy, complete removal of pathological tissue, root-end resection, and establishment of a hermetic apical seal. Resection of approximately 3 mm of the root apex has been shown to eliminate the majority of apical ramifications and lateral canals that may harbor residual microorganisms. Furthermore, a root-end bevel angle approaching 0° minimizes the number of exposed dentinal tubules and reduces the potential for microleakage. While larger bevel angles (30°-45°) improve access, a 0° root-end bevel is currently preferred because it preserves root structure, reduces exposed dentinal tubules, and enhances the apical seal [12].

Mineral trioxide aggregate was selected as the root-end filling material because of its favorable biological and physical properties. MTA exhibits excellent sealing ability, biocompatibility, bioactivity, and the capacity to induce hard tissue formation. Numerous clinical studies have reported high success rates when MTA is used during surgical endodontic procedures, making it one of the most extensively investigated root-end filling materials [13].

Laser-assisted debridement was employed as an adjunctive measure during surgery. The use of diode lasers in periapical surgery has been associated with improved hemostasis, reduction of bacterial load, and enhanced visibility within the surgical field. Improved control of intraoperative bleeding facilitates precise surgical manipulation and may contribute to favorable postoperative healing [14]. In the present case, laser-assisted curettage allowed efficient decontamination of the surgical site while maintaining adequate visualization throughout the procedure.

A distinctive aspect of this case was the incorporation of regenerative techniques to enhance the healing of the extensive osseous defect. Platelet-rich fibrin represents a second-generation platelet concentrate containing leukocytes, cytokines, and growth factors embedded within a fibrin matrix. These biological mediators contribute to angiogenesis, cellular migration, osteoblastic differentiation, and soft tissue maturation. Previous studies have demonstrated accelerated tissue repair and improved bone regeneration when PRF is used in periapical surgery [15].

The combination of PRF with xenograft particles to create sticky bone offers additional advantages by improving graft stability and reducing particulate migration. The fibrin network acts as a biological scaffold that facilitates cellular infiltration and vascularization while maintaining close adaptation of the graft material within the defect. This approach has gained increasing popularity in oral regenerative procedures because of its handling characteristics and regenerative potential [16].

Guided tissue regeneration was additionally utilized to optimize healing. Large periapical defects associated with cortical plate loss are susceptible to invasion by rapidly proliferating epithelial and connective tissue cells, which may interfere with bone regeneration. GTR membranes act as physical barriers that promote selective repopulation of the defect by osteogenic and periodontal ligament cells. Several systematic reviews have reported enhanced healing outcomes when regenerative membranes are used in conjunction with bone graft materials for the management of extensive periapical lesions [17].

Histopathological examination of the excised tissue revealed chronic nonspecific inflammation. Histopathological analysis remains an important component of surgical endodontic treatment because radiographic appearance alone cannot reliably differentiate among granulomas, cysts, and other pathological entities. Submission of all surgically removed tissue for microscopic examination is therefore recommended to establish a definitive diagnosis and exclude uncommon pathologies [18].

The favorable postoperative outcome observed in this patient may be attributed to several factors, including thorough orthograde disinfection, adequate apical sealing with MTA, complete surgical debridement, laser-assisted disinfection, and the use of biologically active regenerative materials. The young age of the patient may also have contributed to the healing response because pediatric and adolescent patients generally demonstrate greater regenerative potential and bone turnover compared with older individuals [19,20].

This case highlights the importance of a multidisciplinary and biologically driven approach when managing persistent periapical pathology in young patients. The integration of contemporary surgical principles with regenerative adjuncts can facilitate preservation of natural dentition and promote predictable healing even in lesions associated with significant cortical bone destruction.

## Conclusions

Persistent periapical lesions that fail to resolve following conventional root canal treatment may require surgical intervention for successful management. This case demonstrates that apicectomy combined with laser-assisted debridement, platelet-rich fibrin, sticky bone grafting, and guided tissue regeneration can result in favorable healing outcomes and preservation of natural dentition. Comprehensive diagnosis using CBCT and the integration of regenerative techniques may enhance the predictability of surgical endodontic treatment in adolescent patients.
